# miR-378-mediated glycolytic metabolism enriches the Pax7^Hi^ subpopulation of satellite cells

**DOI:** 10.1186/s13619-022-00112-z

**Published:** 2022-04-02

**Authors:** Hu Li, Lin Kang, Rimao Wu, Changyin Li, Qianying Zhang, Ran Zhong, Lijing Jia, Dahai Zhu, Yong Zhang

**Affiliations:** 1 The Max-Planck Center for Tissue Stem Cell Research and Regenerative Medicine, Bioland Laboratory (Guangzhou Regenerative Medicine and Health Guangdong Laboratory), Guangzhou, 510005 China; 2 The State Key Laboratory of Medical Molecular Biology, Institute of Basic Medical Sciences, Chinese Academy of Medical Sciences and School of Basic Medicine, Peking Union Medical College, Beijing, 100005 China; 3 Department of Endocrinology, the Second Clinical Medical College of Jinan University, Shenzhen People’s Hospital, Shenzhen, 518020 China

**Keywords:** satellite cells, muscle regeneration, miRNAs, glycolytic metabolism

## Abstract

Adult skeletal muscle stem cells, also known satellite cells (SCs), are a highly heterogeneous population and reside between the basal lamina and the muscle fiber sarcolemma. Myofibers function as an immediate niche to support SC self-renewal and activation during muscle growth and regeneration. Herein, we demonstrate that microRNA 378 (miR-378) regulates glycolytic metabolism in skeletal muscle fibers, as evidenced by analysis of myofiber-specific miR-378 transgenic mice (TG). Subsequently, we evaluate SC function and muscle regeneration using miR-378 TG mice. We demonstrate that miR-378 TG mice significantly attenuate muscle regeneration because of the delayed activation and differentiation of SCs. Furthermore, we show that the miR-378-mediated metabolic switch enriches Pax7^Hi^ SCs, accounting for impaired muscle regeneration in miR-378 TG mice. Mechanistically, our data suggest that miR-378 targets the Akt1/FoxO1 pathway, which contributes the enrichment of Pax7^Hi^ SCs in miR-378 TG mice. Together, our findings indicate that miR-378 is a target that links fiber metabolism to muscle stem cell heterogeneity and provide a genetic model to approve the metabolic niche role of myofibers in regulating muscle stem cell behavior and function.

**Supplementary Information:**

The online version contains supplementary material available at 10.1186/s13619-022-00112-z.

## Background

Skeletal muscle fitness is an important determinant of health and disease. Muscle disease and weakness can be observed under either physiological or pathological conditions, which are accompanied by fiber-type switching and loss of muscle stem cells (Bentzinger and Rudnicki, 2014; Chakkalakal et al., 2012; Li et al., 2019; Schiaffino and Reggiani, 2011). The fiber type composition determines the muscle endurance, strength, and fatigability (Schiaffino and Reggiani, 2011). Skeletal muscle stem cells, also known as satellite cells (SCs), are essential for muscle regeneration and homeostasis (Collins et al., 2005; Yin et al., 2013). Since satellite cells reside between the basal lamina and the muscle fiber sarcolemma, the crosstalk between satellite cells and muscle fibers is important for satellite cell function (Collins et al., 2005; Evano and Tajbakhsh, 2019; Li et al., 2019). In particular, myofibers secrete myokines to modify satellite cell niche. A better understanding of the niche role of myofibers helps to prevent and treat skeletal muscle loss associated with these diseases.

Satellite cells remain quiescent under resting conditions but become activated during muscle regeneration. Pax7, a paired-box transcription factor, plays critical roles in regulating satellite cell functions during development, and Pax7-positive (Pax7^+^) satellite cells are essential for adult skeletal muscle regeneration after skeletal muscle injury (Buckingham and Relaix, 2015; Relaix et al., 2005). More intriguingly, satellite cells are highly heterogeneous, and levels of Pax7 expression contribute to the heterogeneity of satellite cells in mice. Recently, two subpopulations of satellite cells, Pax7^Hi^ and Pax7^Lo^, were identified based on the levels of Pax7 expression in satellite cells (Rocheteau et al., 2012). Interestingly, each of the two subpopulations of satellite cells has unique biological features in mice. Pax7^Hi^ cells have a lower metabolic status than Pax7^Lo^ cells. We recently showed the metabolic niche role of muscle metabolism in regulating Pax7 SC heterogeneity in mice. Pax7^Hi^ cells are dramatically reduced in aged mice, and this aged-dependent loss of Pax7^Hi^ cells is metabolically mediated by myofiber-secreted granulocyte-colony stimulating factor (G-CSF), as Pax7^Hi^ SCs are replenished by exercise-induced G-CSF in aged mice (Li et al., 2019). The findings suggest that manipulation of myofiber metabolism to regulate Pax7^Hi^ cells may represent a potential strategy for treating age-related muscle loss (e.g., sarcopenia) or muscular dystrophy.

MicroRNAs (miRNAs) are endogenous small noncoding RNAs that regulate target gene expression post-transcriptionally (Bartel, 2009; Zhang et al., 2016a). The discovery of miRNAs has provided new insights into the mechanisms that control multiple cellular processes, including fiber metabolism and fiber type composition (Gan et al., 2013; Liu et al., 2016). However, whether miRNAs link myofiber metabolism and muscle stem cell function is largely unknown. miR-378 is located in the first intron of its host gene peroxisome proliferator-activated receptor gamma coactivator-1β (PGC1β) (Gagan et al., 2011; Kang et al., 2020; Podkalicka et al., 2020). The functions of miR-378 have been implicated in mitochondrial metabolism and systemic energy homeostasis (Carrer et al., 2012). Interestingly, miR-378 was highly expressed in glycolytic muscle(Kang et al., 2020; Zeng et al., 2016). We recently showed that global overexpression of miR-378 significantly enhanced glycolytic metabolism in myofibers and delayed satellite cell activation and differentiation (Zeng et al., 2016). In the present study, we used muscle-specific miR-378 transgenic (TG) mice to show that miR-378 regulates glycolytic metabolism and significantly enriches the Pax7^Hi^ subpopulation of satellite cells.

## Results

### miR-378 regulates glycolytic metabolism in skeletal muscle in mice

We have recently reported that transgenic mice with global overexpression of miR-378 exhibit a lean phenotype (Zhang et al., 2016b). One possible mechanism has been proposed: miR-378 stimulates glycolysis in skeletal muscle by activating the pyruvate- -phosphoenolpyruvate futile cycle to maintain energy homeostasis in response to high-fat diet stress (Zhang et al., 2016b). To ascertain the functional roles of miR-378 in regulating skeletal muscle metabolism, we further generated myofiber-specific miR-378 transgenic (TG) mice that expressed miR-378 driven by the promoter of human skeletal alpha-actin (HSA), which drives gene expression in the differentiated muscle cells. Thus, the transgene overexpression is achieved in the differentiated myotubes and mature myofibers (Fig. S1A). In the two examined lines of miR-378 TG mice, miR-378 was specifically overexpressed in skeletal muscle myofibers (tibialis anterior, TA) but not in muscle stem cells (satellite cells, SCs) or in other tissues (e.g., white adipose tissue, WAT) (Fig. S1B). Compared with their wild-type (WT) littermates, miR-378 TG mice had no overt histological and physiological abnormalities in skeletal muscle during development (Fig. S1C-H).

**Fig. 1:**
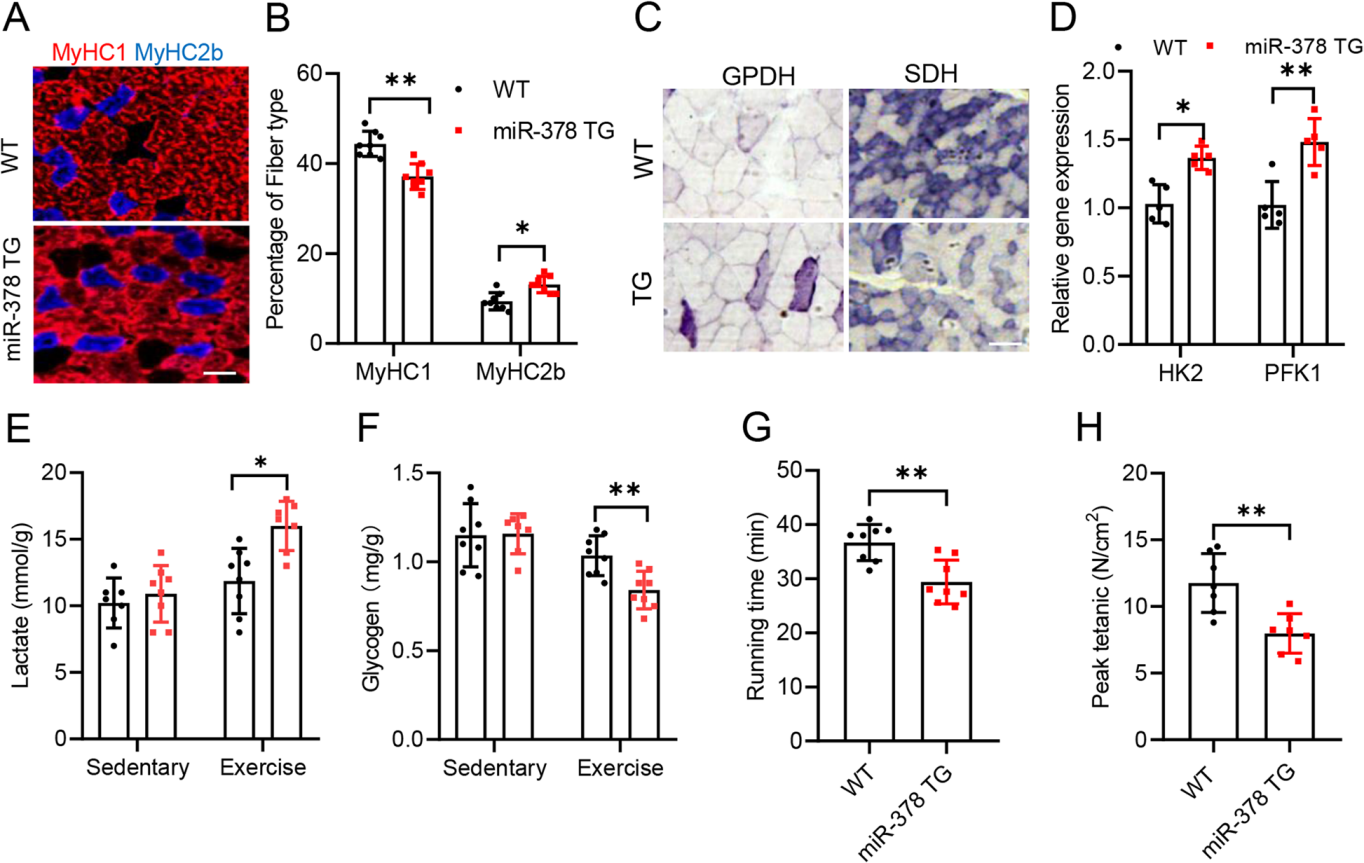
miR-378 regulates glycolytic metabolism of myofibers in mice. **A** Frozen sections of soleus muscle from WT and miR-378 TG mice were immunostained for different myosin heavy chain isoforms (*n* = 8 mice per group). **B** Quantification of immunofluorescence data shown in (**A**) expressed as the mean percentage of total muscle fibers. **C** Representative histochemical staining of α-GPDH (left) and SDH (right) enzymatic activity in gastrocnemius muscle from WT and TG mice (*n* = 8 mice per group). Scale bar, 20 μm. **D** Expression of *HK2* and *PFK1* represented glycolytic metabolic genes in soleus muscle from the indicated genotypes (*n* = 8 mice per group). **E** Tibialis anterior muscle lactate content in running time-matched mice (*n* = 8 mice per group). **F** Tibialis anterior muscle glycogen content in running time-matched mice (*n* = 8 mice per group) **G** Running time of WT and miR-378 TG mice at exhaustion (*n* = 8 mice per group). **H** Peak tetanic force of EDL muscle in the miR-378 TG and wild-type mice (*n* = 8 mice per group)

Next, we determined whether fiber type and metabolism might be altered in myofiber-specific miR-378 TG mice. Immunostaining of myosin heavy chain (MHC) on cryosections of soleus muscle demonstrated an increase in MyHC2b-positive (type II) fibers but a reduction in MyHC1-positive (type I) fibers (Sawano et al., 2016) in 8-week-old miR-378 TG mice compared to WT littermates (Fig. 1A-B). Consistent with the immunostaining results, we observed an increase in type IIb form of MHC (Myh4) at both the mRNA (Fig. S2A) and protein levels (Fig. S2B). In addition, glycolytic activity was also significantly higher in the miR-378 TG mice, as characterized by histochemical staining of α-glycerophosphate dehydrogenase (α-GPDH) as a marker for glycolytic fibers and succinate dehydrogenase (SDH) as a marker for oxidative fibers (Fig. 1C and Fig. S2C). Moreover, the increased glycolytic activity was further supported by augmented expression of the genes encoding two key glycolytic enzymes, hexokinase (*HK2*) and phosphofructokinases (*PFK1*) (Fig. 1D), in the miR-378 TG mice. To explore the physiological consequence of the increased glycolytic activity in myofiber-specific miR-378 TG mice, a forced treadmill exercise test was performed with miR-378 TG and WT littermates. In response to acute exercise training, the levels of muscle lactate (Fig. 1E) were significantly higher, but the muscle glycogen content (Fig. 1F) was lower in the TG mice than in the WT littermates. As a consequence of this increased glycolytic activity, the TG mice exhibited a significantly reduced capacity for running (Fig. 1G) and peak tetanic force (Fig. 1H), indicating the decreased endurance capability in the TG mice. We conclude from these studies that miR-378 is sufficient to activate a program of molecular, metabolic and contractile changes characteristic of fast-twitch glycolytic myofibers.

### Skeletal muscle regeneration is significantly delayed in myofiber-specific miR-378 TG mice

Our recent findings demonstrate that glycolytic metabolism of myofibers favors satellite cell self-renewal instead of satellite cell activation and differentiation (Li et al., 2019). In contrast, Lepper’ group have shown the increased satellite cell pool in oxidative myofibers (Southard et al., 2016). Given myofiber-specific overexpression of miR-378 elevated glycolytic activity, the miR-378 TG mice might provide an additional model to investigate metabolic niche roles of myofibers for satellite cell function and muscle regeneration. To this end, we evaluated the muscle regeneration of the miR-378 TG mice. Muscle damage was induced by cardiotoxin (CTX) injury in the TA muscle of two TG mouse lines and WT littermates, and similar results were observed in both TG mouse lines; therefore, data from the A line are presented in this study.

**Fig. 2:**
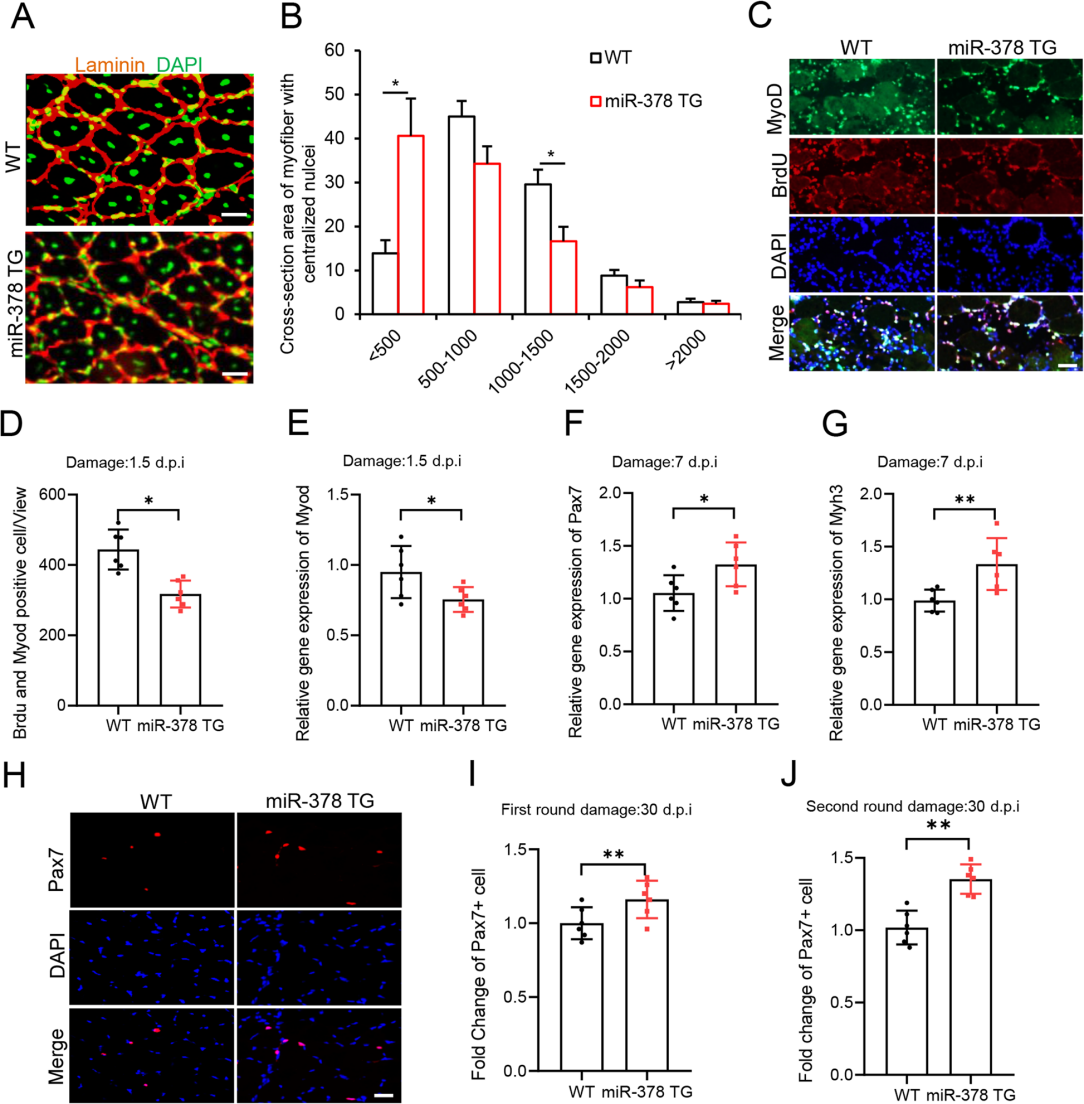
Delayed muscle regeneration in miR-378 transgenic mice. **A** Frozen sections from the TA muscle of miR-378 TG and WT mice damaged for 7 days were immunostained for laminin (red) and DAPI (green) (*n* = 6 mice per group). **B** The cross-sectional area of regenerated myofibers with centralized nuclei was calculated based on the immunostaining in panel (**A**). y axis represents percentage of myofibers with centralized nuclei. **C** Representative views of MyoD (green) and BrdU (red) staining on sections of TA muscle 1.5 days post-CTX injury. DAPI (blue) served to visualize nuclei. **D** Number of MyoD and BrdU double positive cells calculated in TA muscle sections described in panel (**C**). **E** Relative mRNA level of *MyoD* in TA muscle 1.5 days post injury from the miR-378 TG mice and WT littermates, determined by RT-qPCR. **F-G** Relative mRNA levels of *Pax7* (**F**) and *Myh3* (**G**) in TA muscle 7 days post injury from the miR-378 TG mice and WT littermates, determined by RT-qPCR. **H** Representative images of immunostained Pax7 on cryosections of CTX-damaged TA muscles from the miR-378 TG mice and WT littermates 30 days post injury. Scale bars, 50 μm. **I** Number of Pax7 positive cells calculated on TA sections described in panel (H), were normalized to WT controls which were set up to 1. **J** Normalized Pax7 positive cell number in TA muscle at 30 days after the second round of CTX-induced injury. *P < 0.05, **P < 0.01. Two-tailed Student’s t-test was used for all statistical tests

First, we measured the size of regenerating myofibers characterized by centralized nuclei and found that the size of the regenerating myofibers was significantly smaller in miR-378 TG mice than in WT controls 7 days post CTX injury (Fig. 2A-B), indicating delayed muscle regeneration in the TG mice. Therefore, we next examined satellite cell behavior and function in the miR-378 TG mice during muscle regeneration. Satellite cell activation was evaluated by immunostaining for MyoD and BrdU at 1.5 days post injury (Fig. 2C). We found significantly fewer MyoD and BrdU double positive cells in miR-378 TG mice than in WT controls (Fig. 2D) and reduced the level of *MyoD* mRNA in the TG muscle (Fig. 2E), indicating that overexpression of miR-378 in myofibers delayed satellite cell activation during muscle regeneration. At 3.5 days post injury, we observed higher level of *Pax7* mRNA and lower level of embryonic form of myosin heavy chain (*eMHC*, also known *Myh3*) mRNA in damaged TA muscle of the TG mice than that in WT control (Fig. S2D and S2E), supporting delayed myogenesis in the miR-378 TG mice. Subsequently, we found significantly more *Pax7* (Fig. 2F) and *Myh3* (Fig. 2G) mRNA in the damaged muscles of the miR-378 TG mice 7 days post injury, demonstrating that neonatal myofibers formed slower in TG mice than in WT controls. To explore the capacity of self-renewal, we determined the number of Pax7-positive (Pax7^+^) cells at 30 days post CTX damage when muscle regeneration was generally complete. Remarkably, TG mice had more Pax7^+^ satellite cells than wild-type control mice after the first round (Fig. 2H-I) and the second round of muscle regeneration (Fig. 2J). Together, our morphological and molecular analysis data show that muscle regeneration in the miR-378 TG mice was remarkably delayed due to defects in satellite cell activation and differentiation and prone to self-renewal during muscle regeneration.

### miR-378-mediated muscle metabolism fine-tunes Pax7^Hi^ subpopulation cells

**Fig. 3:**
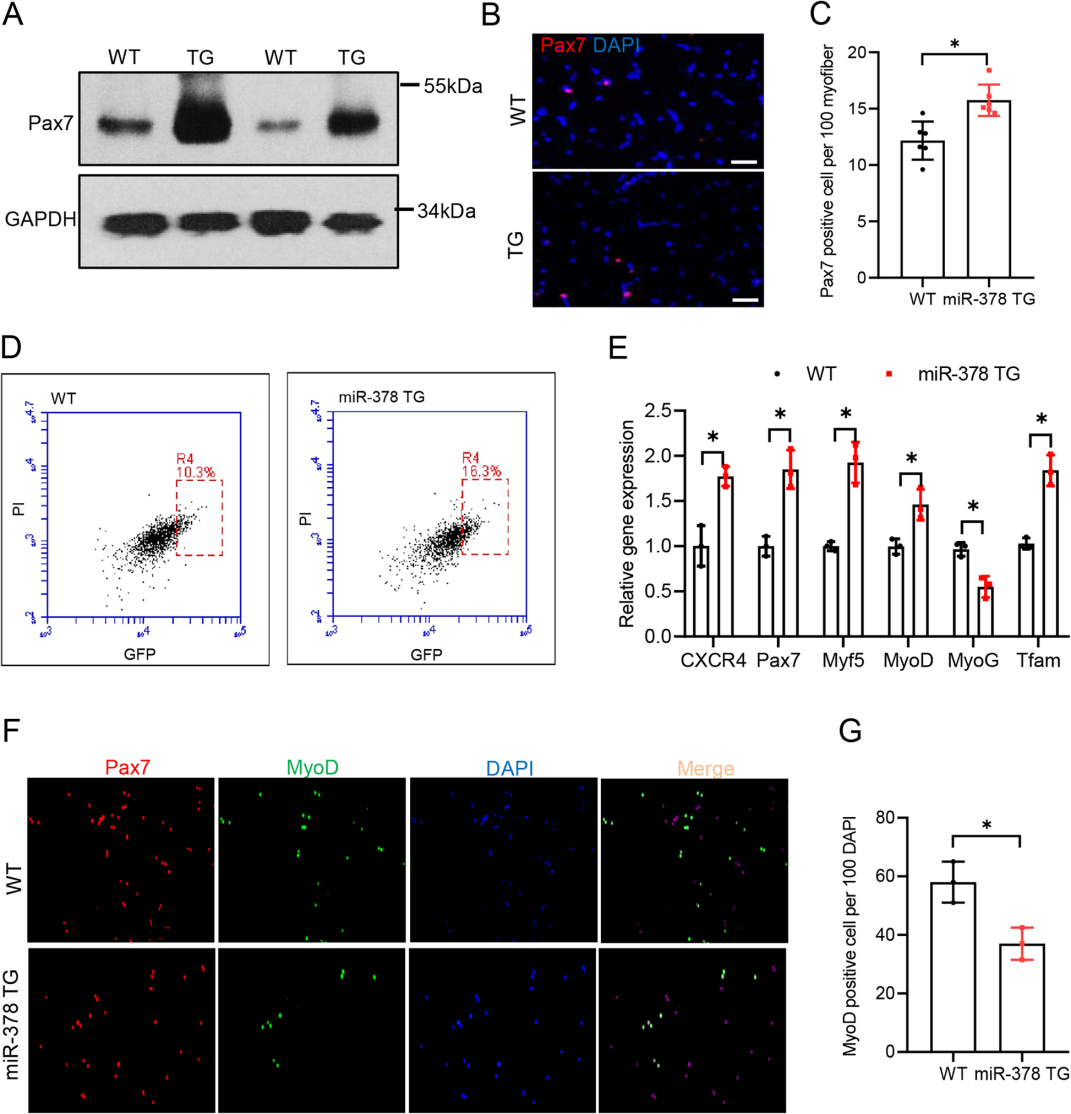
Enriched Pax7^Hi^ subpopulation of satellite cells in miR-378 TG mice.** A** Pax7 protein in TA muscle of miR-378 TG and WT mice was detected by western blot. GAPDH was used as a loading control. **B** A representative view of Pax7 (red) staining on sections of TA muscle from the miR-378 TG mice and WT littermates. DAPI (blue) served to visualize nuclei. Scale bars, 50 μm. **C** Number of Pax7-positive cells in the immunostained sections described in (**B**). **D** The percentage of the Pax7^Hi^ subpopulation was calculated by FACS. **E** Expression of stemness-, differentiation- and mitochondria-related markers determined using real-time RT–PCR in sorted satellite cells from miR-378 TG and WT mice. **F** Satellite cells from the *Pax7-nGFP*;miR-378 TG mice and *Pax7-nGFP* controls were sorted using FACS, cultured for 24 h in growth medium and immunostained for MyoD (red). DAPI (blue) served to visualize nuclei. **G** Numbers of MyoD positive cells calculated in panel (**F**). Values are the means ± s.e.m. of triplicate experiments. *P < 0.05, **P < 0.01. Two-tailed Student’s *t*-test was used for all statistical tests

We have previously reported that glycolytic myofibers, as a metabolic niche, enrich Pax7^Hi^ subpopulation cells (Li et al., 2019). The inhibitory effect of myofiber-specifically overexpressed miR-378 on satellite cell activation prompted us to examine Pax7 expression in the satellite cells of the TG mice. First, we observed significantly more Pax7 protein in TA muscle from both TG mouse lines than WT mice (Fig. 3A). Immunostaining of Pax7 indicated that the number of satellite cells was slightly but significantly higher in the TG mice than in WT control mice (Fig. 3B-C). However, the slightly more number cannot explain such significant more Pax7 protein level in the miR-378 TG mice. Given the higher levels of Pax7 expression in TA muscle of miR-378 TG mice, we tested whether satellite cells in miR-378 TG mice were enriched in the Pax7^Hi^ subpopulation. To directly test this possibility, we sorted satellite cells from skeletal muscle of *Pax7-nGFP*;miR-378-TG mice and *Pax7-nGFP* as controls, and the percentage of Pax7^Hi^ and Pax7^Lo^ subpopulations was analyzed in the sorted satellite cells (Rocheteau et al., 2012). We found that the Pax7^Hi^ subpopulation in sorted cells was 6% larger in TG mice than in WT control mice (Fig. 3D and Fig.S2F). In addition, satellite cells from the miR-378 TG mice expressed higher levels of stemness-related genes, including *CXCR4* and *Pax7*, but lower levels of the myogenic differentiation gene *MyoG* and higher level of the metabolism-related gene *Tfam* (Fig. 3E), which are typical features of Pax7-nGFP^Hi^ cells (Li et al., 2019). To characterize the function of Pax7^Hi^ satellite cells in miR-378 TG mice, we assessed their activation capability. Sorted satellite cells from hind limb skeletal muscle of TG and WT control mice were cultured in growth medium for 24 h and immunostained with antibodies against MyoD (Fig. 3F). MyoD^+^ cells in the TG mice were significantly reduced (Fig. 3G), indicating delayed activation of satellite cells in miR-378 TG mice. We also isolated single fibers from extensor digitorum longus (EDL) muscle and cultured ex vivo for 72 h. Immunostaining showed the typical clusters of SC progeny that are proliferating (Pax7^+^/MyoD^+^), differentiating (Pax7^–^/MyoD^+^) and self-renewing (Pax7^+^/MyoD^–^) (Fig. S2G). The SC progeny from miR-378 TG myofibers had significantly more self-renewing cells and significantly fewer differentiating cells than WT controls (Fig. S2H), which are typical features of Pax7^Hi^ cells. Together, our in vivo and ex vivo data suggest that miR-378 TG mice are enriched in Pax7^Hi^ satellite cells.

### miR-378-mediated myofiber metabolic reprogramming by targeting the Akt1-FoxO1 axis

**Fig. 4:**
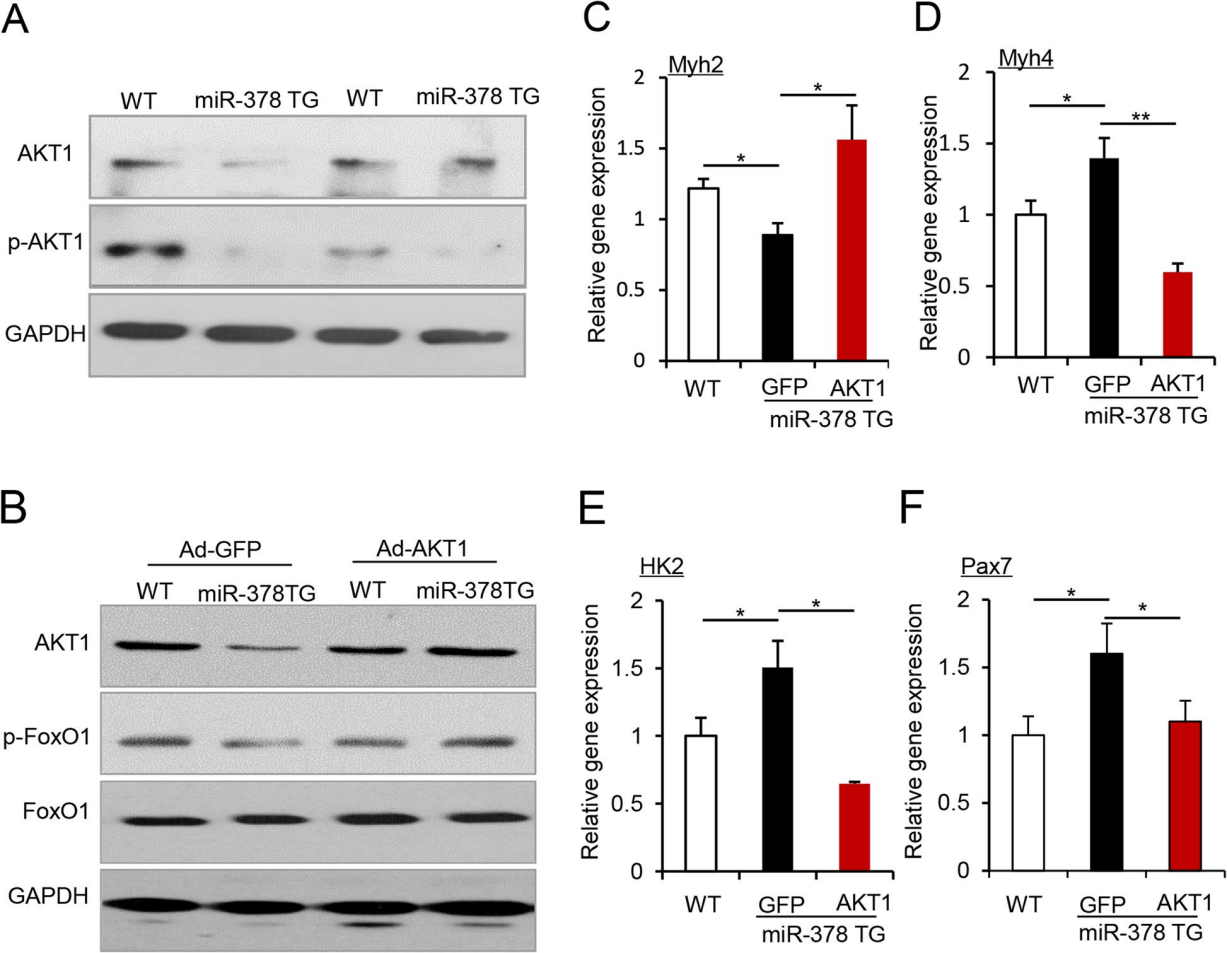
AKT1 is one of the targets of miR-378 in skeletal muscle. **A** Protein levels of AKT1 in TA muscle from WT and the miR-378 TG mice were measured by western blot. GAPDH was used as a loading control. **B** Protein levels of AKT1, p-FoxO1, and FoxO1 in TA muscle intramuscularly injected with adenovirus expressing AKT1 (Ad-AKT1) of the miR-378 TG mice and WT littermates, determined by western blot. Adenovirus solely expressing EGFP (Ad-GFP) served as control. GAPDH was used as a loading control. **C-F** The expression of *Myh2* (**C**), *Myh4* (**D**), *HK2* (**E**), and *Pax7* (**F**) in WT and miR-378 TG mice intramuscularly injected with Ad-AKT1 as described in panel (**B**) (*n* = 3 mice per group)

Next, we investigated the molecular mechanism underlying miR-378-mediated metabolic reprogramming of myofibers by identifying miR-378 targets. Given that we have previously reported that miR-378 activates the pyruvate-PEP futile cycle by targeting Akt1 in skeletal muscle (Zhang et al., 2016b), we examined Akt1 in the miR-378 TG mice and found that Akt1 protein levels were significantly reduced in the muscles of myofiber-specific miR-378 TG mice (Fig. 4A and Fig. S3A), indicating that Akt1 is a miR-378 target in skeletal muscles in mice. FoxO1, downstream of Akt1, plays a critical role in regulating myofiber switching and metabolism. To determine whether miR-378 mediated the metabolic shift by targeting the Akt1-FoxO1 axis, we overexpressed Akt1 in the TA muscle of WT and TG mice by injecting adenovirus expressing Akt1 (Ad-Akt1) (Fig. 4B and Fig. S3B). Akt1 overexpression abolished metabolic reprogramming by miR-378, as indicated by the expression of the *Myh*2 (Fig. 4C) and *Myh*4 (Fig. 4D) and the key enzyme *HK2* (Fig. 4E). Meaningfully, Akt1 overexpression reversed *Pax7* expression in the miR-378 TG mice (Fig. 4F). Since we could not rule out the possibility that some satellite cells were transduced by the adenovirus expressing AKT, the decreased Pax7 level might be not solely attributed by Akt1-mediated alternation of muscle metabolism. Together, our data reveal that miR-378 mediates myofiber metabolic reprogramming by targeting the Akt1-FoxO1 axis, which favors Pax7^Hi^ subpopulation of satellite cells.

## Discussion

In this study, we uncovered miR-378 as a target that linked myofiber metabolism to muscle stem cell heterogeneity. Using myofiber-specific miR-378 TG mice, we demonstrated that miR-378 regulated glycolytic metabolism in skeletal muscle by targeting the Akt1/FoxO1 pathway. Interestingly, myofiber-specific miR-378 TG mice exhibited delayed muscle regeneration due to defective activation of satellite cells. Furthermore, we found that the enriched Pax7^Hi^ cells in myofiber-specific miR-378 TG mice accounted for the delayed activation of satellite cells and attenuated muscle regeneration. Thus, we provide a genetic model to further prove the metabolic nice role of myofibers in regulating muscle stem cell behavior and function.

The physiological functions of miR-378 have been implicated in mitochondrial metabolism, systemic energy homeostasis, classic brown adipose tissue-specific expansion and cancer metabolism (Carrer et al., 2012; Pan et al., 2014; Zhang et al., 2016b). Although globally overexpressing miR-378 transgenic mice display a lean phenotype and resist high-fat diet-induced obesity, likely due to enhanced glycolysis in skeletal muscle tissues (Zhang et al., 2016b), myofiber-specific miR-378 TG mice are still lacking to clarify whether the miR-378-mediated muscle metabolism switch is detrimental or adaptive to whole body metabolic homeostasis. Our experimental system further corroborated that miR-378 regulates glycolytic metabolism in skeletal muscle by targeting the Akt1 pathway.

One of the major findings in our study is that myofiber-specific overexpression of miR-378 delayed the activation of muscle stem cells and muscle regeneration. This finding was consistent with the phenotype of globally overexpressing miR-378 transgenic mice (Zeng et al., 2016). These observations raised an intriguing question of how miR-378 overexpression in myofibers affects muscle stem cell function. One possibility is likely that myofiber-derived miR-378 is secreted into the local niche and directly taken up by satellite cells. To rule out this possibility, we overexpressed miR-378 in satellite cells and observed no overt effect on its activation and differentiation (Fig. S4), suggesting that miR-378 likely does not directly regulate muscle stem cell function. Another possibility might be that the miR-378-mediated metabolic switch influences the muscle secretome profile (cytokines or metabolites), thereby modifying the niche of satellite cells. Identifying such factors would be of great help to understand the metabolic niche role of myofibers in regulating muscle stem cell function. Indeed, we recently identified a muscle-secreted G-CSF that functions as a metabolic niche factor regulating muscle stem cell self-renewal and enriching the Pax7^Hi^ subpopulation of SCs (Li et al., 2019). To support this notion, we found that *G-CSF* expression was increased in myofiber-specific miR-378 TG mice compared to WT controls (Fig. S5). Therefore, such niche factors might play roles as mediator(s) linking muscle metabolism and muscle stem cell function.

## Conclusions

Collectively, our findings highlight the pivotal roles of myofibers as metabolic niches for muscle stem cell function. For physiological relevance, we provide a potential target to manipulate muscle metabolism, which in turn benefits muscle stem cell function to counteract muscular diseases or muscle aging.

## Methods

### Mouse lines and animal care

Myofiber-specific miR-378 transgenic (TG) mice were generated by the Model Animal Research Center of Nanjing University. Two transgenic lines were established from two founders identically created with the same plasmid DNA construct containing miR-378 precursor sequences and the human skeletal muscle actin promoter to drive transgene miR-378 expression (Figure S1A), and sex- and age-matched wild-type littermates served as the control group throughout all experiments presented in the study. *Pax7-nGFP* Tg mice were kindly gifted by Dr. Shahragim Tajbakhsh (Institute Pasteur, France) and crossed with miR-378 transgenic (TG) mice. 8-week-old mice were used in this study. Mice were housed in an animal facility and given free access to water and standard rodent chow. All animal procedures were approved by the Animal Ethics Committee of Peking Union Medical College, Beijing (China).

### Fluorescence activated cell sorting (FACS)

Pax7 SCs from the skeletal muscles of *Pax7-nGFP*;miR-378 TG mice were fluorescently sorted as previously described (Wu et al., 2015). Briefly, mononuclear muscle-derived cells were isolated from the tibialis anterior (TA) muscles of *Pax7-nGFP*;miR-378 TG mice by dispase and collagenase digestion, filtered through 70-µm and 40-µm cell strainers, and directly sorted with a BD Aria II Cell Sorting System. The sorted cells were cultured in growth medium (F-10 Ham’s medium supplemented with 20% FBS, 4 ng/ml basic fibroblast growth factor, and 1% penicillin/streptomycin) on collagen-coated cell culture plates at 37 °C and 5% CO2.

### SDH and GPDH staining

SDH and GPDH staining were performed as previously described (Li et al., 2019). For measurement of succinate dehydrogenase (SDH) activity, muscles were harvested and serial tissue cross-Sects. (10-µm) were cut at -20 °C and adhered to glass coverslips. The coverslips were inverted and placed over a microscope slide reaction chamber. The tissue was first incubated in the dark at 23 °C in a substrate-free blank solution consisting of 1 mM sodium azide, 1 mM l-methoxyphenazinemethosulfate (MPMS), 1.5 mM NBT, and 5 mM EDTA in 100 mM sodium phosphate buffer (pH 7.6). The reaction was allowed to proceed for 10 min to allow the nonspecific staining to plateau. The blank was then replaced with a substrate solution consisting of the above reagents plus 48 mM succinic acid. Images were captured every 3times for 10 min. For measurement of α-glycerophosphate dehydrogenase (α-GPDH) activity, serial Sects. (14-µm) were cut, adhered to glass coverslips, and distributed between two Coplin jars kept at -20 °C. A blank solution consisting of 1 mM sodium azide, 1 mM MPMS, and 1.2 mM NBT in 100 mM sodium phosphate buffer (pH 7.4, 37ºC) was added to one jar while a solution of the above reagents plus 9.3 mM α-glycerophosphate was introduced into the other for the substrate reaction. The tissue sections were incubated in the dark for 24 min at 37 °C, the reactions were stopped by extensive rinsing with distilled water. The images were captured using a microscope (Olympus).

### Treadmill

miR-378 TG mice were subjected to treadmill exercise using an Exer3/6 (Columbus Instruments) as previous described (Li et al., 2019; Wu et al., 2015). Each mouse ran on the treadmill at 20° downhill, starting at a speed of 10 cm/s. After 3 min, the speed was increased by 2 cm/s to a final speed of 20 cm/s. Then the mice were allowed to run 25 min.

### Muscle injury and regeneration

Briefly, 8-week-old mice were anesthetized by intraperitoneal injection of ketamine (10 mg/kg) and xylazine (1 mg/kg), followed by injection of 20 μl of 10 μM cardiotoxin (CTX; Sigma, St Louis, USA) in phosphate-buffered saline (PBS) into the mid-belly of the right tibialis anterior (TA) muscle. As an internal control, 20 μl PBS was injected into the left TA muscle of each mouse. Muscles were harvested 1.5 and 7 days after injection to assess the completion of regeneration and repair.

### Western blot analysis

Skeletal muscle tissues were homogenized and lysed on ice in lysis buffer (50 mM Tris (pH 7.5), 150 mM NaCl, 0.5% Nonidet P-40, and protease inhibitor cocktail). Total proteins from skeletal muscle were resolved by SDS–PAGE and then immunoblotted using primary antibodies against Pax7 (DSHB), Myh4 (BF-F3, DSHB), AKT1 (75,692, CST), p-AKT1 (9018, CST), FoxO1 (2880, CST), p-FoxO1 (9461, CST), and GAPDH (Millipore) overnight at 4 °C. After washing with tris-buffered saline containing 0.1% Tween-20 (TBST) for 30 min, membranes were incubated with horseradish peroxidase-conjugated secondary antibodies (Zhongshanjinqiao Corporation, Beijing, China) for 1 h at room temperature and then washed with TBST for 30 min. Membranes were then incubated for 1 min at room temperature in Detection Solution (Thermo Scientific, Waltham, MA, USA) and subsequently exposed to X-ray film.

### Immunofluorescence

FACS-resolved Pax7 SCs were seeded on collagen-coated glass slides in 24-well plates in growth medium (F-10 containing 20% FBS) for 24 h, fixed with 4% formaldehyde for 5 min, permeabilized in 0.1% Triton-X100 in PBS for 15 min at room temperature, and then blocked with 3% bovine serum albumin for 30 min. The cells were incubated with primary antibodies against MyoD (Santa Cruz, SC-760) overnight at 4ºC. The cells were then washed with PBS containing 0.1% BSA and incubated for 2 h with fluorescein-conjugated secondary antibodies (Zhongshanjinqiao Corporation) and Hoechst or DAPI. After several washes with PBS, the cells were examined under a fluorescence microscope (Olympus).

For cryosections of TA muscle, the slides were incubated in 1.0% Triton X-100 in PBS at room temperature for 10 min. Subsequently, the sections were incubated at room temperature for 1 h in filtered blocking buffer (4% BSA, 0.1% Triton X-100). The primary antibodies were diluted with PBS buffer containing 4% BSA. MyoD (Santa Cruz, SC-760), BrdU (ab6326, abcam), Laminin (ab11575, abcam). MyHC1 (BA-D5) and MyHC2b (BF-F3) were purchased from the Developmental Studies Hybridoma Bank (DSHB). Primary antibodies were loaded onto a specimen and incubated overnight at 4 °C. Then the slides were washed with PBS containing 0.1% BSA and incubated for 1 h with fluorescein-conjugated secondary antibodies (Zhongshanjinqiao Corporation) and Hoechst or DAPI. After several washes with PBS, the samples were imaged under a fluorescence microscope (Olympus).

### Real-time RT–qPCR analysis

Total RNA was extracted from cells using TRIzol reagent (Invitrogen, Grand Island, NY, USA) and reverse-transcribed (RT) using RevertAid reverse transcriptase (Thermo Scientific). To measure the mRNA levels of MyoG and MHC, quantitative PCR (qPCR) analyses were performed with SsoFast EvaGreen Supermix (Bio–Rad, 1,725,201), and GAPDH was used as an internal control.

### Muscle force measurement

Muscle force was measured as described (Wu et al., 2015). In brief, the EDL muscles were constantly immersed in a physiological saline solution containing 118.5 mM NaCl, 4.7 mM KCl, 2.4 mM CaCl2, 3.1 mM MgCl2, 25 mM NaHCO3, 2 mM NaH2PO4, and 5.5 mM D-glucose. All solutions were continuously bubbled with 95% O2/5% CO2 (vol/vol) and maintained at pH 7.4. All experiments were carried out at 25 °C. Contractions were elicited by passing a current between two platinum electrodes placed on opposite sides of the muscle. Tetanic contractions were elicited with a 200-ms train of the same pulse at 200 Hz. Contractions were elicited every 2 min during the experiment. Muscle length was adjusted to obtain maximum tetanic force, and a 30-min equilibrium period was allowed before force-frequency measurements. Force was measured with a dual-mode muscle lever system (Grass X88 stimulator) and digitized at 5 kHz with an analog–digital board (Grass). The peak twitch force and peak tetanic force were calculated as the difference between the maximum force during contraction and the force measured 5 m sec before the contraction.

### Statistical analysis

The results are presented as means ± s.e.m. The statistical analyses were performed with Student’s *t*-tests. A *p value* < 0.05 was considered to represent a statistically significant difference.

## Supplementary Information

**Additional file 1.** (TIF 1473 kb)

**Additional file 2.** (TIF 2909 kb)

**Additional file 3.** (TIF 330 kb)

**Additional file 4.** (TIF 1336 kb)

**Additional file 5.** (TIF 156 kb)

## Data Availability

All data generated or analyzed in the present study are included in this published article and the supplementary material. Requests for materials should be addressed to the corresponding author.

## Abbreviations

HK2: Hexokinase 2
PFK1: Phosphofructokinase 1
MyoD: MiR-378, myogenic differentiation 1
CXCR4: C-X-C motif chemokine receptor 4
Myh3: Myosin heavy chain 3
Myh2: Myosin heavy chain 2
Myh4: Myosin heavy chain 4
*MyoG*: Myogenin
α-GPDH: α-Glycerophosphate dehydrogenase
SDH: Succinate dehydrogenase
