# Correction to: miR-378-mediated glycolytic metabolism enriches the Pax7Hi subpopulation of satellite cells

**DOI:** 10.1186/s13619-022-00117-8

**Published:** 2022-04-22

**Authors:** Hu Li, Lin Kang, Rimao Wu, Changyin Li, Qianying Zhang, Ran Zhong, Lijing Jia, Dahai Zhu, Yong Zhang

**Affiliations:** 1 The Max-Planck Center for Tissue Stem Cell Research and Regenerative Medicine, Bioland Laboratory (Guangzhou Regenerative Medicine and Health Guangdong Laboratory), Guangzhou, 510005 China; 2 The State Key Laboratory of Medical Molecular Biology, Institute of Basic Medical Sciences, Chinese Academy of Medical Sciences and School of Basic Medicine, Peking Union Medical College, Beijing, 100005 China; 3 Department of Endocrinology, the Second Clinical Medical College of Jinan University, Shenzhen People’s Hospital, Shenzhen, 518020 China

**Correction to: Cell Regeneration 11, 11 (2022)**

**https://doi.org/10.1186/s13619-022-00112-z**

Following publication of the original article (Li et al., 2022), due to a typesetting error, the sentence below was missing in the article.

Hu Li and Lin Kang contributed equally to this work.

The correct information has been provided in this Correction and the original article (Li et al., 2022) has been updated.
