# Supplementary figures and images for: miR-378-mediated glycolytic metabolism enriches the Pax7Hi subpopulation of satellite cells
Source: Cell Regen. 2022 Apr 2;11:11. doi: 10.1186/s13619-022-00112-z (PMC8976867; doi:10.1186/s13619-022-00112-z)

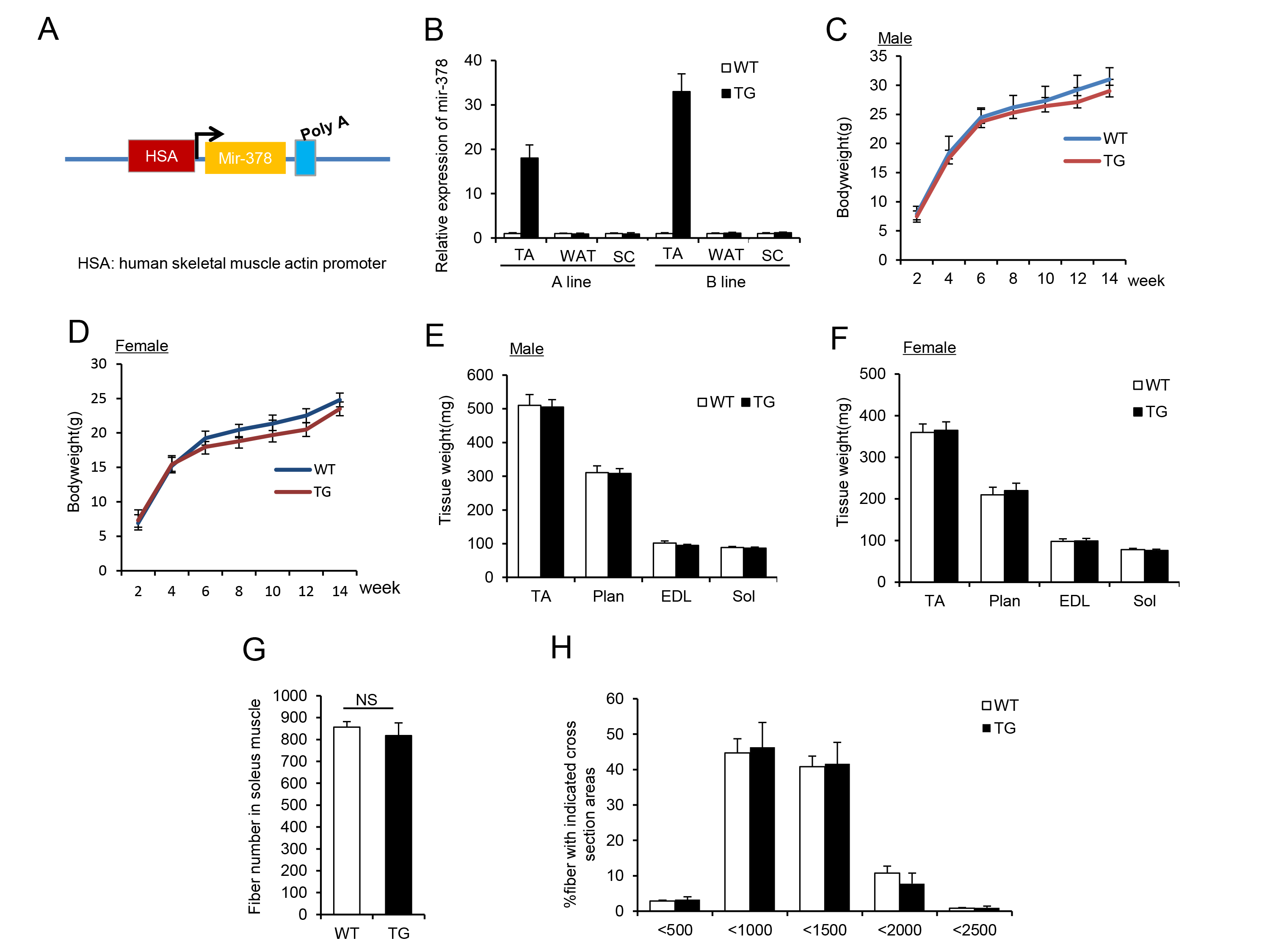

Supplement: Supplementary file 1 — Additional file 1. (TIF 1473 kb) [file 13619_2022_112_MOESM1_ESM.tif]

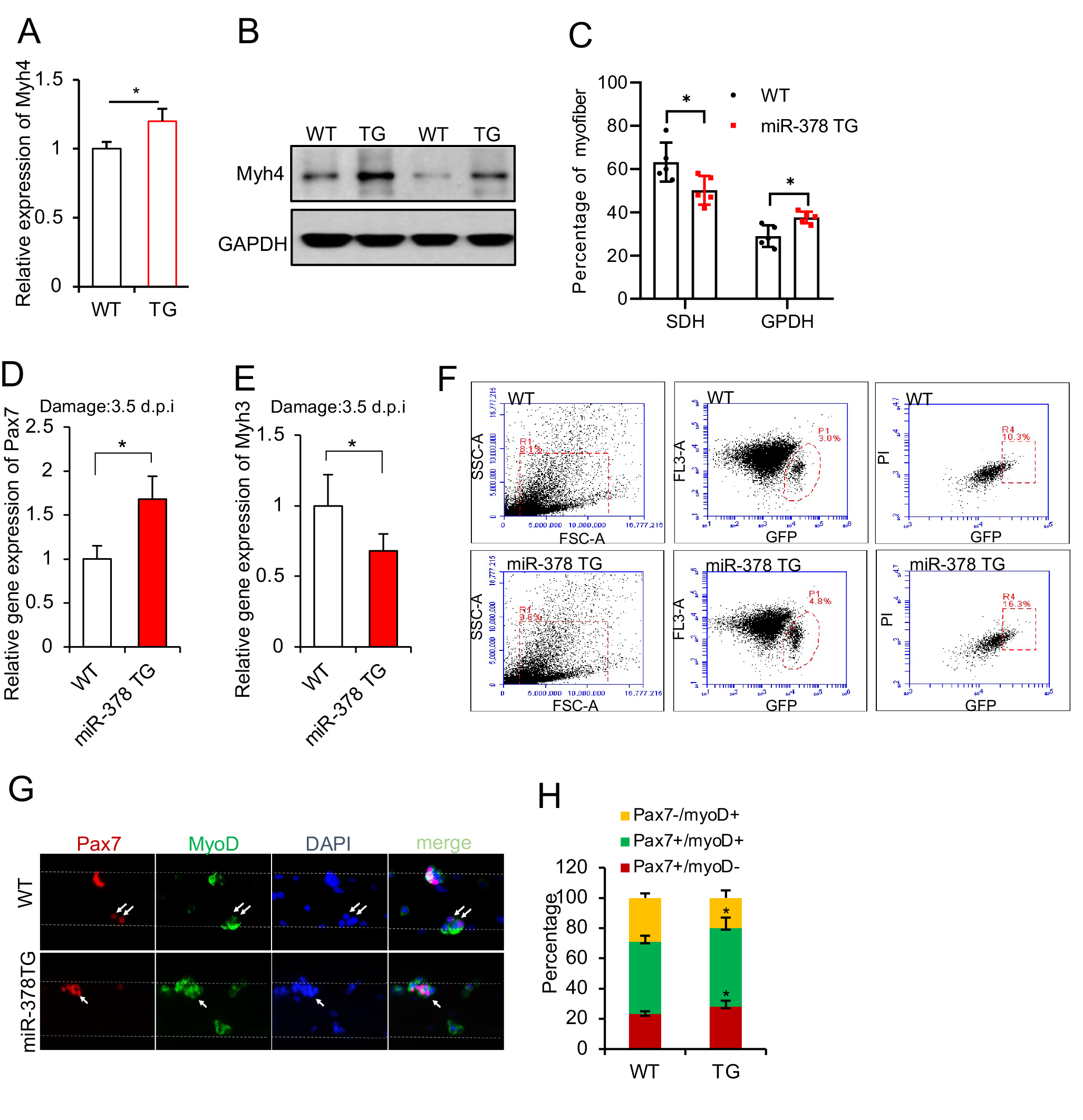

Supplement: Supplementary file 2 — Additional file 2. (TIF 2909 kb) [file 13619_2022_112_MOESM2_ESM.tif]

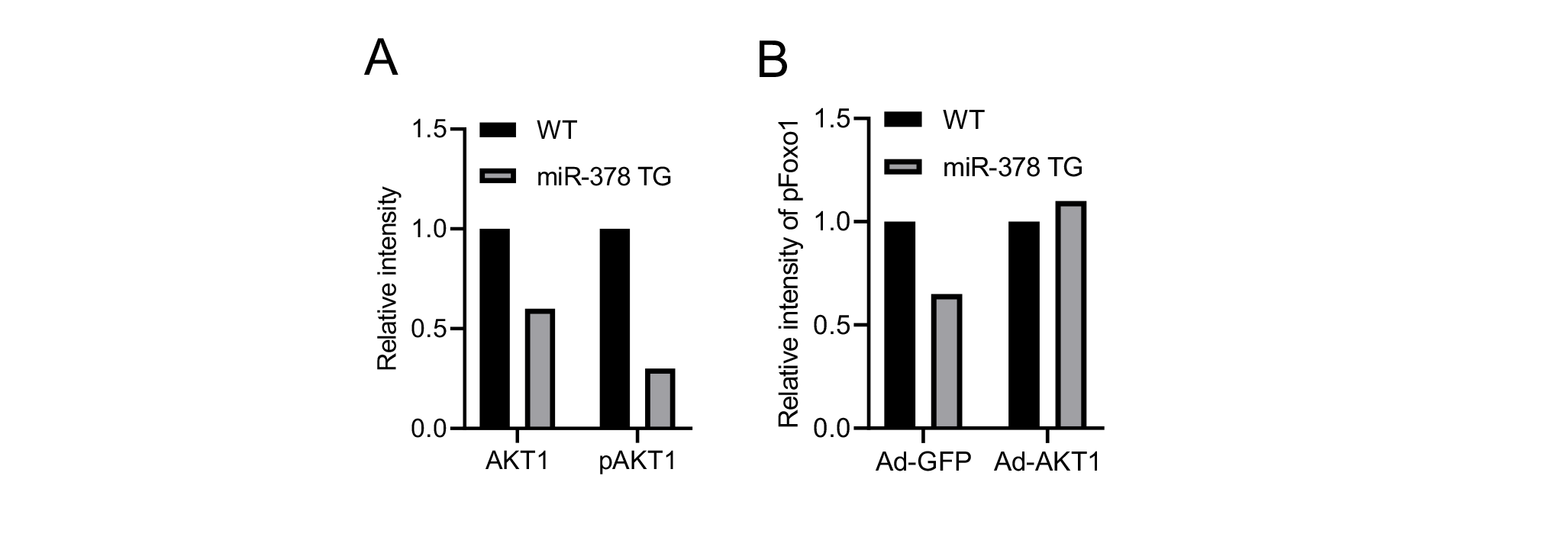

Supplement: Supplementary file 3 — Additional file 3. (TIF 330 kb) [file 13619_2022_112_MOESM3_ESM.tif]

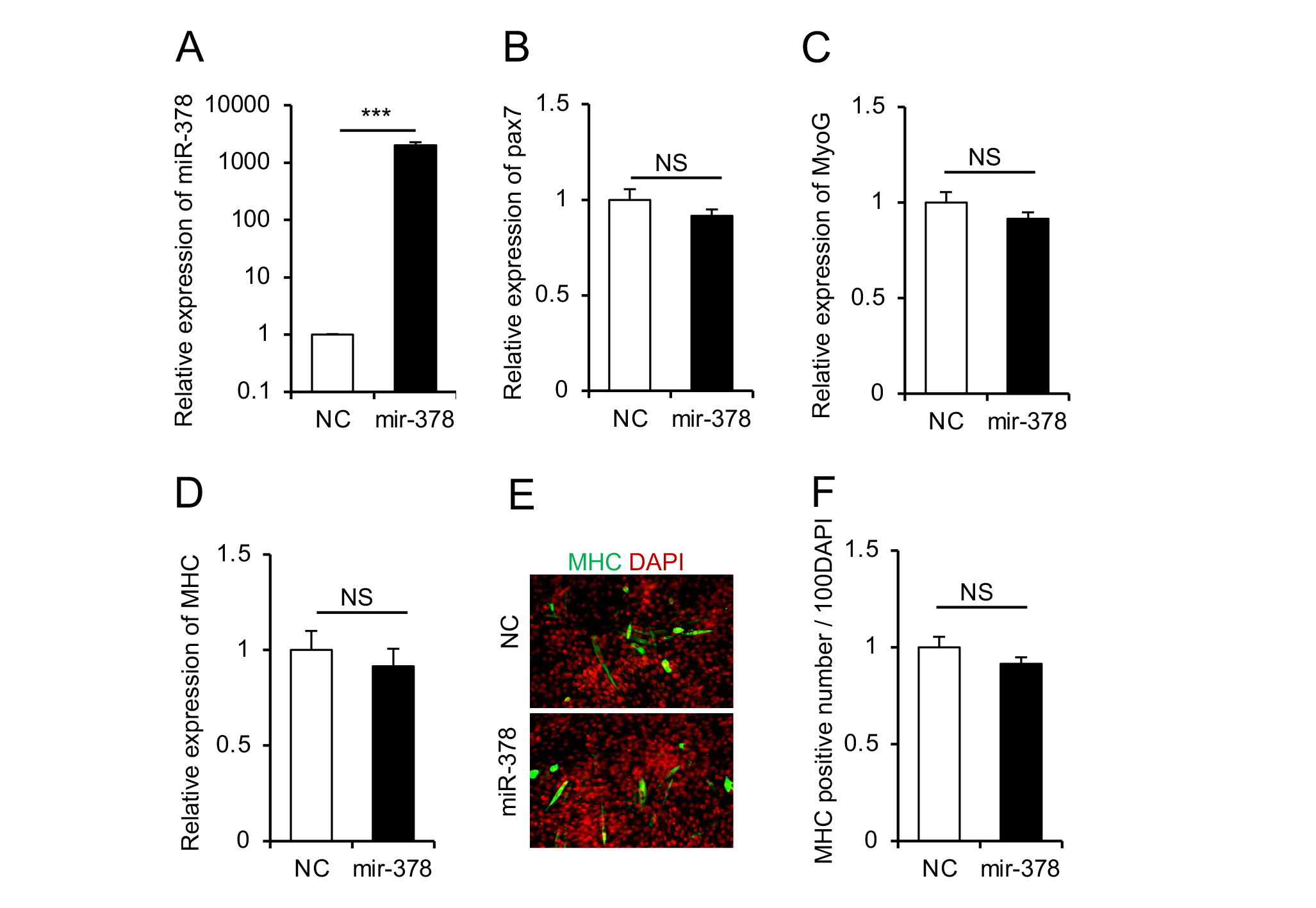

Supplement: Supplementary file 4 — Additional file 4. (TIF 1336 kb) [file 13619_2022_112_MOESM4_ESM.tif]

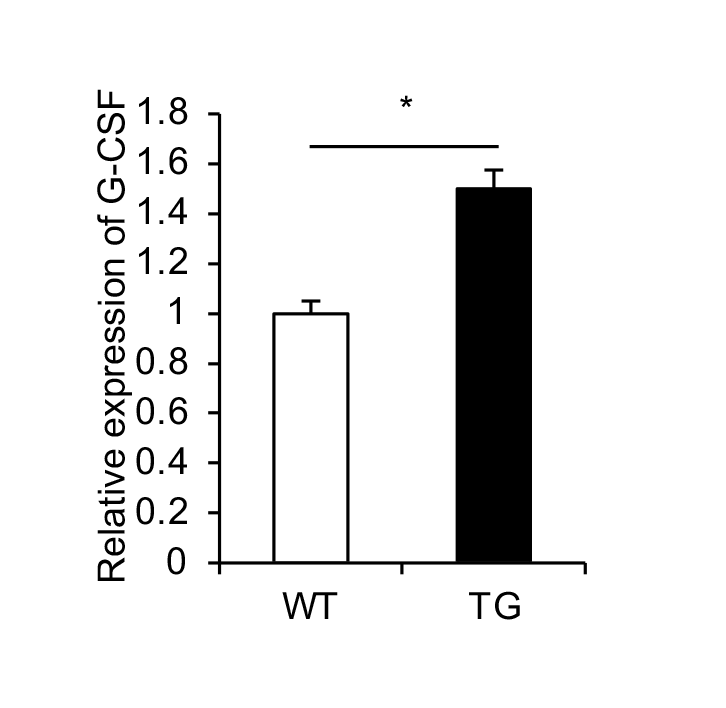

Supplement: Supplementary file 5 — Additional file 5. (TIF 156 kb) [file 13619_2022_112_MOESM5_ESM.tif]
